# Modulation of miRNA Signature in Human Adipose Tissue After 3 Months of ω-3PUFA Supplementation

**DOI:** 10.3390/cells15070577

**Published:** 2026-03-25

**Authors:** James Hernandez, Matthew Lee, Mary Cochran, Ting Li, Panwen Wang, Dawn K. Coletta, Cassandra Rau, Valentin Dinu, Eleanna De Filippis

**Affiliations:** 1 Research Department, Mayo Clinic Arizona, Scottsdale, AZ 85259, USA; hernandez.james1@mayo.edu (J.H.);; 2 Genomic and Computational Biology, Center of Biomedical Research, University of Pennsylvania, Philadelphia, PA 19104, USA; matthew.lee1@pennmedicine.upenn.edu; 3 Barrett, The Honors College, Arizona State University, Tempe, AZ 85281, USA; 4 Department of Health Sciences Research, Mayo Clinic Arizona, Scottsdale, AZ 85259, USA; wang.panwen@mayo.edu; 5 Department of Medicine, College of Medicine, University of Arizona, Tucson, AZ 85724-5017, USA; dcoletta@arizona.edu; 6 College of Health Solutions, Arizona State University, Tempe, AZ P.O. Box 879020, USA; 7 Endocrinology Division, Mayo Clinic Arizona, Scottsdale, AZ 85259, USA

**Keywords:** obesity, insulin resistance, inflammation, human studies, omega 3 PUFA, miRNA, adipose tissue

## Abstract

**Highlights:**

**What are the main findings?**

- Twelve weeks of ω-3PUFA treatment alters AT miRNA expression in individuals with obesity and insulin resistance.
- AT expression of miR-4498 and miR-5689 increases after 12 weeks of ω-3PUFA supplementation.

**What is the implication of the main finding?**

- AT miRNA may have a potential role in the regulation of inflammatory and metabolic pathways.
- The elucidation of AT miRNA’s role in inflammatory and metabolic pathways may provide a potential therapeutic target.

**Abstract:**

Obesity is a persistent public health issue, often resulting in metabolic complications such as insulin resistance (IR). The secretion of pro-inflammatory cytokines from adipose tissue (AT) is increased during obesity, contributing to the impairment of systemic insulin sensitivity. While interventions in animal models have shown that reducing inflammation restores insulin sensitivity, human studies reducing systemic inflammation have produced inconsistent results. We recently demonstrated that three months of high-dose (4 g/daily) ω-3PUFA (fish oil, FO) supplementation improved insulin sensitivity, and decreased systemic and AT inflammation in individuals with obesity (BMI  ≥  30 kg/m^2^). Given recent studies highlighting the involvement of microRNA (miRNA) in inflammatory cytokine production, we investigated the effect of ω-3PUFA supplementation on AT miRNA expression in this cohort. AT biopsies were collected before and after ω-3PUFA supplementation. miRNA was processed on the Affymetrix miRNA 4.0 GeneChip and analyzed using existing inflammatory gene sets sourced from MSigDB. Unbiased, differentially expressed miRNA analysis identified miR-4498 and miR-5689 as significantly increased after three months of ω-3PUFA supplementation. Real-time PCR confirmed bioinformatic analysis findings. Our study reports the modulation of miRNA in AT, reductions in systemic and AT markers of inflammation, and the improvement of IR post ω-3PUFA supplementation. Further research is needed to elucidate the link between miR-4498, miR-5689, and whole-body insulin sensitivity.

## 1. Introduction

Obesity is a complex condition characterized by the excessive accumulation of adipose tissue (AT) resulting from genetic, environmental, and socioeconomic factors, which can have significant impacts on an individual’s health [1]. With one third of the world’s population now affected by obesity, it has become a global public health concern of pandemic proportions [2]. Comorbidities associated with obesity are disabling and life-threatening; these include, but are not limited to, insulin resistance (IR), type 2 diabetes mellitus, cardiovascular disease, and obesity-related cancers [2]. Mortality data analyses have shown that obesity and its complications are associated with a reduced lifespan [3,4].

Obesity causes AT to undergo hypertrophy and hyperplasia in response to the increased storage of nutrients, shown as an increase in the cellular size and number of adipocytes [5]. These changes in AT are accompanied by alterations in the immune cell population in AT and the dysregulation of cytokine secretion [6]. As a result, a chronic, low-grade inflammatory state that negatively affects adipocyte function develops, AT function is impaired, and IR is established [6]. Although therapeutic options to improve IR are expanding, they are still suboptimal. In animal models, treatments that reduce inflammation, such as ω-3 polyunsaturated fatty acid (ω-3PUFA) supplementation, have shown promise in improving inflammation and reducing IR [7].

ω-3PUFAs—are known to have anti-inflammatory properties, reducing markers such as Interleukin-1 (IL-1), Interleukin-6 (IL-6), macrophage chemoattractant protein-1 (MCP-1), and C-reactive protein (CRP), while also decreasing triglyceride concentrations [8]. Fish consumption and/or fish oil (FO) supplementation are effective sources of **ω**-3 polyunsaturated fatty acids (ω-3PUFAs) [9]. FO is a preferred treatment option due to its safety, accessibility, and cost-effectiveness [10]. Although studies in animal models have demonstrated the effectiveness of ω-3PUFA in reducing inflammation and improving IR, inconsistent results have been reported in human interventions [7]. Various factors, such as dosage, duration of intervention, subject characteristics, and measures of inflammation and insulin sensitivity, may contribute to the discrepancy [7]. However, our recent study has demonstrated that supplementation with high-dose ω-3PUFA for 3 months improves insulin sensitivity and reduces inflammation in subjects with obesity and IR [11]. A better understanding of the role of ω-3PUFA in inflammation and IR may lead to the identification of new drug targets for treatment of these obesity-related diseases. The underlying mechanisms of these effects are currently being investigated.

Animal studies have recently revealed docosahexaenoic acid (DHA), an ω-3PUFA found in FO, produces anti-inflammatory effects in animal models through the modulation of microRNA (miRNA) [12]. These molecules are single-stranded noncoding RNAs which act as negative post-transcriptional regulators [13]. miRNA exerts its effect by silencing translation or by destabilizing the target mRNA [14,15]. Increased miRNA expression leads to higher rates of destabilization, resulting in decreased protein expression [16]. Studies in animal models of diet-induced obesity (DIO) have reported increased expression of miRNAs involved in glucose metabolism, resulting in impaired glucose tolerance in those animals [17,18]. Similarly, miRNAs associated with inflammation and IR are found elevated in people with obesity [19,20]. miRNAs are also known to regulate numerous cellular processes and exhibit tissue-specific expressions [21]. The dysregulation of many miRNAs which have direct roles in regulating adipogenesis, adipose differentiation, and insulin secretion is observed during obesity [22]. Moreover, adipocytes are known to be a key source of circulating miRNA that regulate metabolic homeostasis [23].

Our study investigated the effect of ω-3PUFA supplementation on the miRNA signature in individuals with obesity and IR. Our patients showed reduced inflammation and improved insulin sensitivity. We assessed the gene expression of miRNA isolated from subcutaneous (SC) AT biopsy collected before and 3 months after 4 g/day of ω-3PUFA supplementation. We hypothesize for high-dose supplementation of ω-3PUFA to upregulate miRNA expression and differentially regulate the expression of miRNAs controlling metabolic and/or inflammatory gene expression.

## 2. Materials and Methods

### 2.1. Subjects

A detailed description of the ω-3PUFA supplementation study can be found in our previous manuscript [11]. Briefly, twelve nondiabetic participants, 9 women and 3 men between the age 18–65 years, non-smokers, with BMI ≥ 30 kg/m^2^, were recruited via advertisement at Mayo Clinic Arizona, local newspapers, and the internet (www.clinicalconnection.com or https://clinicaltrials.gov/ct2/show/NCT02378077) [11]. All studies were conducted in the Clinical Studies Infusion Unit (CSIU) at the Mayo Clinic Arizona and were adherent to the ethical standards of this institution. This study has been reviewed by and received approval from the Mayo Clinic IRB and registered at www.clinicaltrials.gov (NCT02378077). After informed, written consent was obtained, anthropometric data was collected and screening laboratory testing was completed. Body composition was assessed by Bioimpedance Analyzer (BIA310, Biodynamics Corporation, Shoreline, WA, USA). All participants completed a 75 g oral glucose tolerance test (OGTT) to confirm absence of diabetes mellitus according to the American Diabetes Association criteria. Subjects returned the next day to complete an 80 mU/m^2^ min euglycemic–hyperinsulinemic clamp to assess baseline insulin sensitivity. The study participants were then dispensed a 1-month supply (120 g) of Nature Made Burp-less Fish Oil capsules, 500 mg per capsule (Nature Made, West Hills, CA, USA) (Lot# 2217828) to be ingested at 4 g/day with meals. Patients returned monthly for clinical assessment, discussed adherence to study parameters, collected blood samples to assess free fatty acid plasma levels, and were given a new supply of fish oil capsules. After 12 weeks ω-3 PUFA supplementation, a second euglycemic–hyperinsulinemic clamp was administered to assess post-intervention insulin sensitivity. AT biopsies were obtained pre- and post-supplementation as per study protocol and are described below [11].

### 2.2. Subcutaneous Adipose Tissue Biopsy

On a subsequent visit, an SC fat biopsy was extracted, in a sterile fashion, as we previously described [11]. Fasting subjects had 3–5 g of tissue removed from the lower abdominal area, beneath the navel and above the pubic area [11]. Whole tissue AT was used to isolate mRNA for further evaluation. This procedure was repeated after 12 weeks after ω-3 PUFA supplementation.

### 2.3. miRNA Isolation and RT-PCR Analysis

miRNAs were isolated using the miRNeasy Mini Kit (Qiagen Germantown, MD, USA), according to the manufacturer’s protocol. The miScript PCR System, consisting of MISCRIPT II RT KIT (50) and miScript SYBR Green PCR Kit (Qiagen Germantown, MD, USA), was then used to convert miRNA to cDNA and for real-time PCR analysis (RT-PCR), respectively. Both kits were used according to the manufacturer’s protocol. RT-PCR analysis was performed using the CFX384 Touch RT-PCR Detection System (Bio-Rad Hercules, CA, USA) with the primers listed in Table 1, and our internal control gene was β-actin.

### 2.4. Microarray Analysis

RNA integrity and concentration was assessed by Agilent Bioanalyzer (Agilent Scientific Instruments, Santa Clara, CA, USA) prior to sample preparation. Microarray analysis was performed according to the manufacturer’s instructions using the FlashTag™ Biotin HSR kit (ThermoFisher, Waltham, MA, USA). Briefly, total RNA was incubated with ATP and Poly A polymerase to add a 3′ polyA tail. Ligation followed, employing a dT bridging oligo to covalently attach a multiple-biotin molecule containing a 3DNA dendrimer to the miRNA population. Labeled samples were subsequently processed on the Affymetrix miRNA 4.0 GeneChip™ (ThermoFisher, Waltham, MA, USA) according to the manufacturer’s instructions. Chips were hybridized using 100 μL solution at 48 °C for 16 h. GeneChips were washed and stained with streptavidin–phycoerythrin and scanned using the Affymetrix 3000 7G scanner (ThermoFisher, Waltham, MA, USA). All Affymetrix array experiments were carried out at the Mayo Clinic Genome Analysis Core. Control parameters were confirmed to be within normal ranges before normalization and data reduction was initiated.

### 2.5. Differentially Expressed miRNA

The cell intensity files were generated from the stored images that contain a single intensity value for each probe cell on the array. After normalization, the expression values obtained were submitted for analysis with linear models of microarray data (LIMMA v3.38.3). To correct for multiple testing, *p*-values were adjusted using the Benjamin and Hochberg method. Genes with false discovery rate < 5% and fold change > 1.5 were classified as significantly differentially expressed. Gene function annotation and enrichment analysis were performed using the Database for Annotation, Visualization, and Integrated Discovery (DAVID) [24]. We used Enrichr [25] and GSEA [26] for pathway enrichment analysis.

### 2.6. Gene Set Analysis

Four different groups of gene sets were used to explore differentially expressed genes in pre- and post-treated samples. The first three groups include Gene Ontology biological processes, Gene Ontology cellular component, and Gene Ontology molecular functions. The last group of gene sets were configured by drawing any gene set in MSigDb [27] that contained the word “inflammation”. Gene set variation analysis (GSVA) was conducted on our samples using each of the four groups. GSVA identifies enrichments in a sample-to-sample independent manner. Following GSVA, Student’s *t*-test was conducted to identify differences in gene set enrichment between our pre- and post-treated samples. Using a *p*-value threshold of 0.01, four separate lists (corresponding to the four initial groups of gene sets) of gene sets that were significantly different between our treatment groups were curated. Then, using the enrichment scores, unsupervised hierarchical clustering using the complete linkage method was performed.

## 3. Results

Before starting ω-3 PUFA supplementation, participants demonstrated the presence of systemic inflammation, as determined by the elevated levels of plasma inflammatory markers, including C-reactive protein (CRP) [11]. We previously noted that 12 weeks of high-dose ω-3 PUFA supplementation significantly decreased plasma pro-inflammatory cytokines MCP-1, IL-1B, INFy, GM-CSF, and TNFa [11]. As previously reported [11], after ω-3 PUFA supplementation we detected an improvement in glucose disposal by 26% during euglycemic–hyperinsulinemic clamp. This improvement represented an improvement in insulin resistance without changes in total body weight, fat mass, or body mass index (BMI) (Table 2) [11].

### 3.1. Comprehensive Gene Set Enrichment Analysis Results

Our study employed gene set variation analysis (GSVA) to uncover the molecular mechanisms responsible for the observed differences between pre- and post-treatment groups. GSVA is a powerful computational method that identifies classes of genes which are over/under-represented in large gene sets and may have an association with disease phenotypes. This approach allows us to gain insights into the biological processes, pathways, and cellular mechanisms affected by the treatment [28,29].

### 3.2. Immunity-Related and Longevity Gene Set Analysis

**Figure 1:**
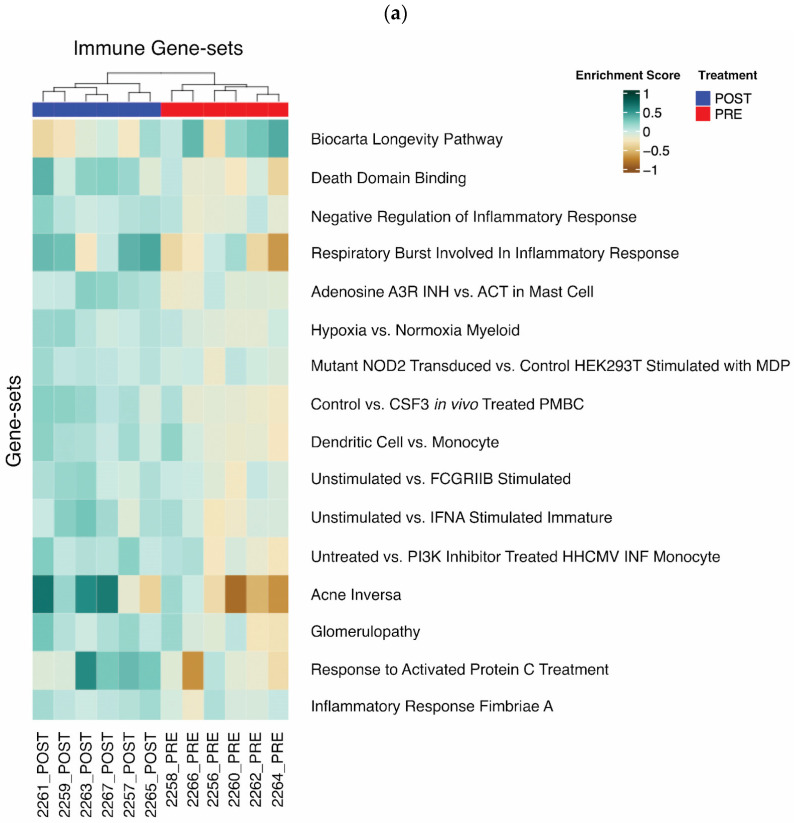

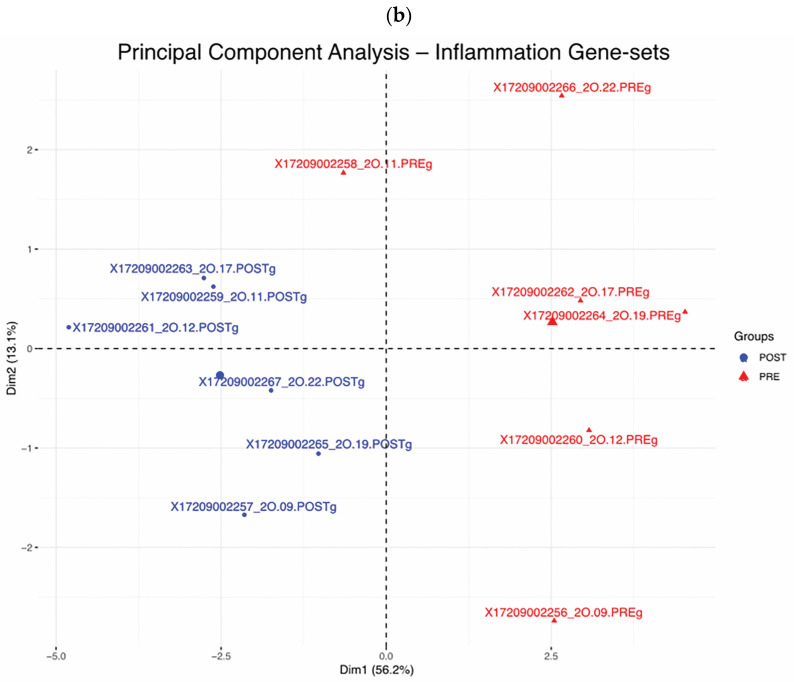
Differential expression of immunity-related gene sets after 12 weeks of ω-3PUFA supplementation. GSVA followed by Student’s *t*-test identified 12 immune-related gene sets from the molecular signatures database (MSigDB) significantly different between pre- and post-treatment samples using a *p*-value threshold of 0.01. (**a**) Unsupervised clustering (Euclidean distance metric) with the 16 immunity-related gene sets from the molecular signatures database (MSigDB). The pre/post samples clearly separate into their respective groups. (**b**) Principal component analysis (PCA), a form of dimensionality reduction for visualization, applied to the 16 significant gene sets from panel (**a**). The first two principal component plots clearly differentiate between the pre- and post-treatment groups.

We began our analysis by examining enrichments for all immunity-related gene sets in the molecular signatures database (MSigDB). Through differential enrichment analysis using Student’s *t*-test, we identified 16 gene sets that were statistically different (*p* < 0.01) between the pre- and post-treatment groups (Figure 1a). These findings suggest a significant impact of the treatment on immune-related processes. Many studies have previously shown that a reduction in AT inflammation has a beneficial effect on metabolism. Unsupervised clustering of the samples revealed distinct grouping by treatment status (Figure 1a), further supporting the robust effect of the treatment on immune-related gene expression patterns. This clear separation indicates that the treatment induces a consistent and measurable change in the expression of immunity-related genes across samples. Notably, we observed enrichment of the gene set “Negative Regulation of Inflammatory Response” in our post-treatment samples. This finding is in accordance with other markers of reduced inflammation detected at the plasma levels (MCP-1, IL-1B, INFγ, GM-CSF, and TNFα) observed after treatment with ω-3PUFA. A suppression of the “Negative Regulation of Inflammatory Response” clearly supports that our treatment has an anti-inflammatory effect in AT during obesity and IR. Additionally, 3 months of ω-3PUFA supplementation in patients with obesity led to the enrichment of genes in the “Biocarta Longevity Pathway” which is regulated through the IGF-1 receptor [30] and may be a result of reduced inflammation after ω-3PUFA supplementation. Dimensionality reduction in the statistically significant differentially enriched gene sets further stratified samples by treatment group (Figure 1b). This visualization reinforces the distinct immunological states induced by the treatment and provides a clear representation of the global shifts in immune-related gene expression patterns.

### 3.3. Gene Ontology Biological Processes Analysis

**Figure 2:**
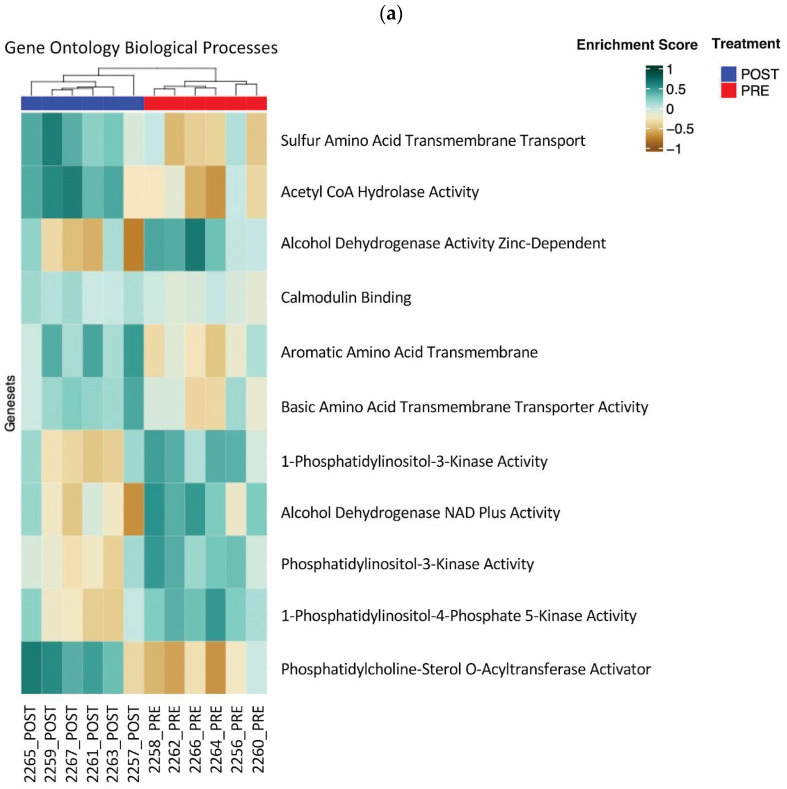

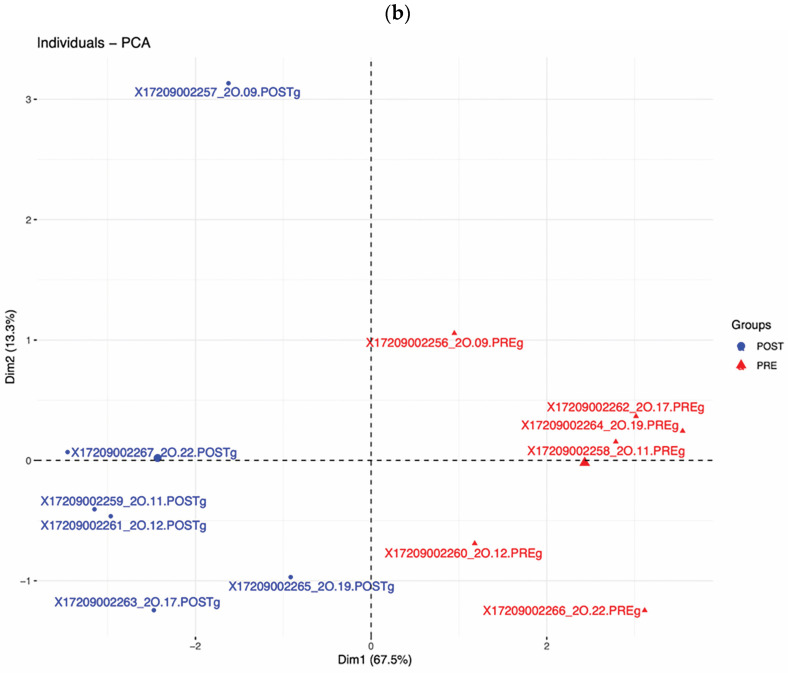
Differential expression of biological process gene sets after 12 weeks of ω-3PUFA supplementation. GSVA followed by Student’s *t*-test identified 11 GO biological process gene sets significantly different between pre- and post-treatment samples using a *p*-value threshold of 0.01. (**a**) Unsupervised clustering with the 11 GO biological process gene sets. The pre/post samples clearly separate into their respective groups. (**b**) PCA applied to the 11 gene sets from panel (**a**). The first two principal component plots clearly differentiate between the pre- and post-treatment groups.

Expanding our investigation beyond immunity-specific processes, we analyzed Gene Ontology (GO) biological processes gene sets. This analysis yielded a similar stratification of pre- and post-treatment groups, indicating that the treatment’s effects extend to broader biological functions. Using the previously described statistical approach, we identified 11 differentially enriched gene sets (*p* < 0.01) (Figure 2a). Of note, genes associated with acetyl-CoA hydrolase activity have been enriched. Elevated levels of acetyl-CoA, the substrate for this enzyme, are linked to IR and AT inflammation [31]. Acetyl-CoA disrupts normal metabolic pathways and interferes with insulin’s ability to effectively regulate glucose uptake. Reduction in acetyl-CoA through enhanced acetyl-CoA hydrolase activity may be a factor in the decreased inflammatory status and improved insulin sensitivity seen in our cohort after ω-3PUFA supplementation. Of further interest, several gene sets involved in amino acid transport are shown to be enriched after ω-3PUFA supplementation. Increased circulating levels of branched-chained amino acids (BCAAs) have been shown to be related to obesity and IR [32]. Sulfur-containing amino acids, methionine and cysteine have also been shown to be associated with obesity and IR where increased cysteine plasma level correlates with increased BMI [33] and methionine correlates with increased BCAA and the risk for development of T2DM [34]. The enrichment of these pathways is notable and may be worthy of further research. Principal component analysis (PCA) using these gene set enrichment scores demonstrated that the first two principal components effectively differentiated our treatment groups (Figure 2b). This clear separation in the PCA plot underscores the treatment’s profound and multifaceted impact on cellular biological processes.

### 3.4. Cellular Component Gene Set Analysis

**Figure 3:**
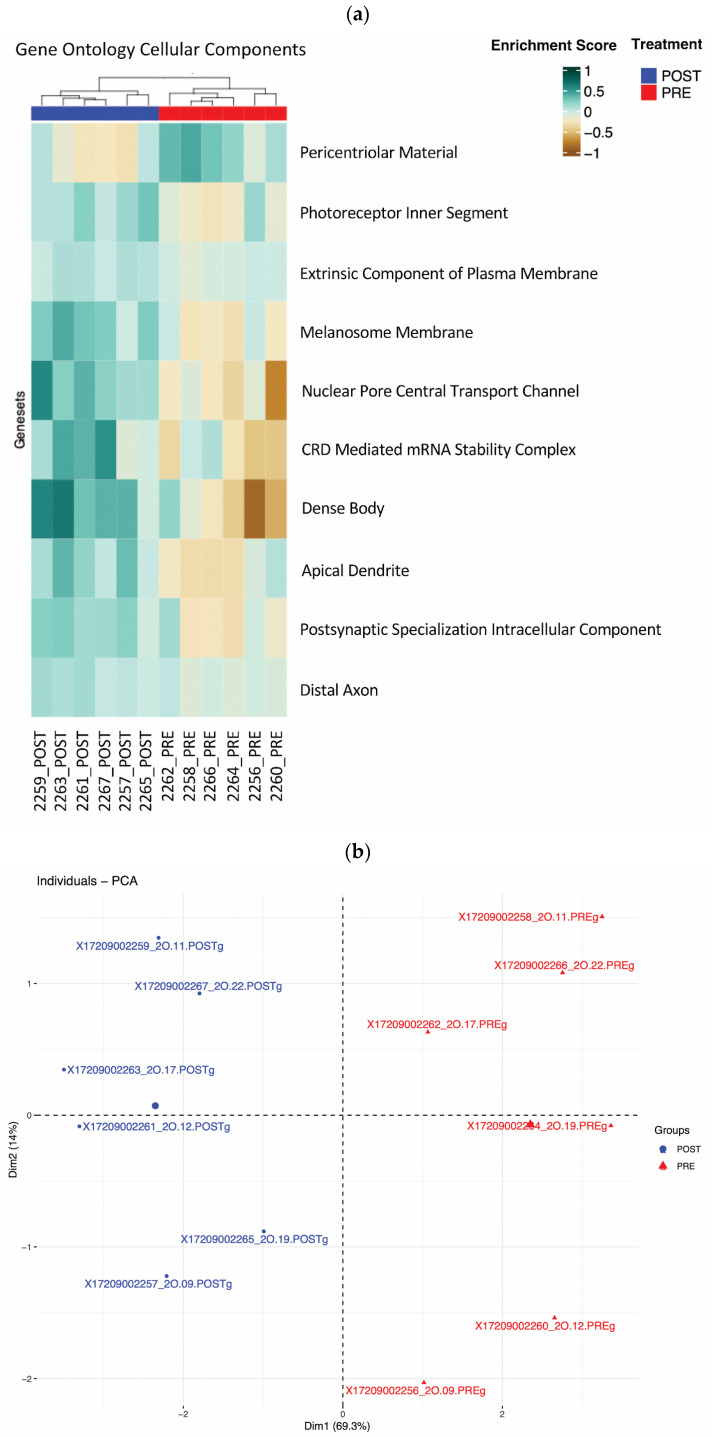
Differential expression of GO cellular component gene sets after 12 weeks of ω-3PUFA supplementation. GSVA followed by Student’s *t*-test identified 10 GO cellular component gene sets which are significantly different between pre- and post-treatment samples using a *p*-value threshold of 0.01. (**a**) Unsupervised clustering with the 10 GO cellular component gene sets. The pre/post samples clearly separate into their respective groups. (**b**) PCA applied to the 10 gene sets from panel (**a**). The first two principal component plots clearly differentiate between the pre- and post-treatment groups.

To gain insights into the subcellular localization of treatment-induced changes, we examined gene sets from different cellular components. This analysis revealed 10 differentially enriched gene sets (*p* < 0.01) that clustered by treatment groups (Figure 3a). These included sets related to cellular dense bodies, the nuclear pore central transport channel, and several membrane component pathway genes. This suggests ω-3PUFA supplementation may induce changes in genes involved in the intra- and extra-cellular movement of macromolecules. The enrichment of these pathways may be involved in the improvement of glucose disposal observed in our cohort. Principal component analysis of these cellular component gene sets showed that the first two components stratified the samples by treatment (Figure 3b). This clear separation emphasizes that the treatment’s effects are manifest across various cellular compartments, suggesting a comprehensive cellular response.

### 3.5. miRNA Dysregulation Analysis

**Figure 4:**
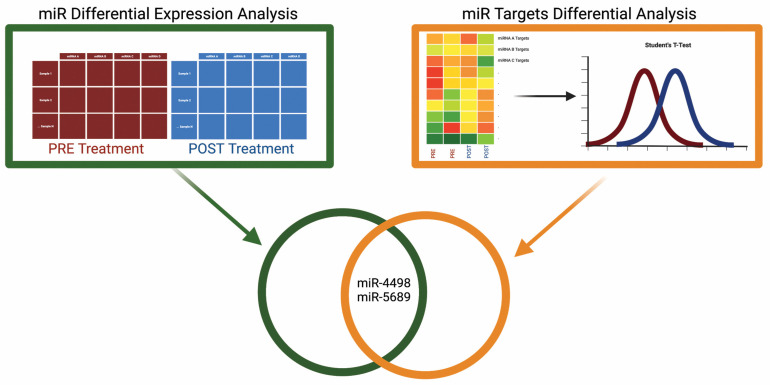
Identification of candidate miRNA regulators. Two-layer analysis where we intersect (**left panel**) the set of differentially expressed miRNAs with (**right panel**) the set of miRNAs whose targeted gene set is differentially expressed between pre- and post-treatment groups. (**left panel**) Differential miRNA expression analysis is performed to identify miRNAs with different expression levels between the pre- and post-treatment groups. (**right panel**) First, GSVA is first performed (heatmap on left) where each miRNA is assigned a gene set formed by the collection of genes targeted by that miRNA. Next, Student’s *t*-test is used to identify miRNA-targeted gene sets that are differentially enriched between pre- and post-treatment groups. Finally, to identify potential regulators, we take the intersection of the sets determined in steps left panel/right panel.

**Figure 5:**
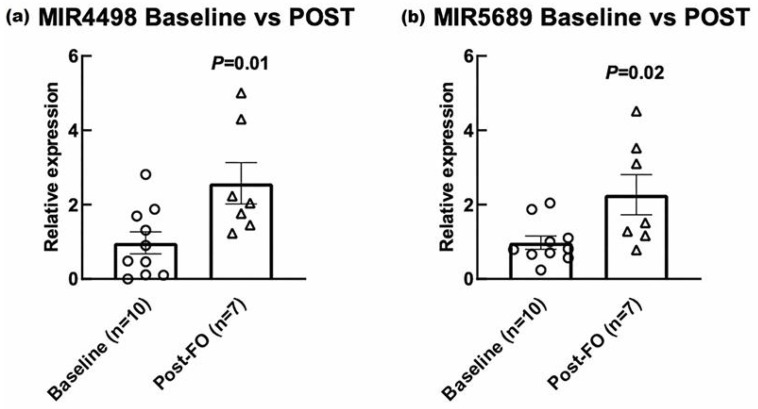
miR-4498 and miR-5689 mRNA expression in human SC-AT pre and post 12 weeks ω-3PUFA supplementation. Quantitative real-time PCR evaluation of SC-AT mRNA shows (**a**) miR-4498 and (**b**) miR-5689 are significantly increased after 12 weeks ω-3PUFA supplementation. Data were analyzed by unpaired 2-tailed *t*-test and expressed as mean ± SEM. *n* = 10 (pre) and 7 (post), respectively.

We next investigated the dysregulation of miRNAs between our treatment groups using a two-step approach. First, we identified potentially dysregulated miRNAs by examining the differential enrichment of mRNA gene sets targeted by specific miRNAs. Student’s *t*-test was conducted to directly identify differentially expressed miRNAs. The intersection of these analyses identified miR-4498 and miR-5689 as significantly dysregulated, along with their target genes (Figure 4). These findings provide insights into the post-transcriptional regulatory changes induced by the treatment. The concurrent dysregulation of these miRNAs and their target genes underscore the complex regulatory networks affected by the treatment, involving both transcriptional and post-transcriptional mechanisms. Lastly, RT-PCR conducted on available AT confirmed increases in both miRNA-4498 and miRNA-5689 in the AT of our cohort following 3 months ω-3PUFA supplementation (Figure 5).

## 4. Discussion

We previously demonstrated that 12 weeks of high-dose (4 g/daily) ω-3PUFA (FO) supplementation in people with obesity (BMI ≥  30 kg/m^2^) decreased systemic and AT inflammation, resulting in increased insulin sensitivity. miRNA levels are known to be altered in people with obesity [35], which is now hallmarked as a state of chronic, low-grade inflammation. In this study, we evaluated miRNA expression in AT biopsies, pre and post ω-3PUFA supplementation, as potentially contributing to the observed improvement in insulin sensitivity. Here, we report that the expression of miR-4498 and miR-5689 were upregulated in the AT of individuals with obesity after undergoing ω-3PUFA supplementation. The gene sets shown to be associated with these miRNAs are involved in diverse physiologic processes affecting inflammation, intercellular biological processes, and even the regulation of cellular components.

Obesity has been reported to decrease life span [36,37]. Our findings suggest that FO can change the regulation of longevity pathways, previously reported to be downregulated in obesity [38]. Interestingly, as shown for improvement in IR indices, the enrichment of longevity pathways in AT occurred without any change in body weight. These results strengthen evidence of the potential use of ω-3PUFA to mediate inflammation and insulin sensitivity as well as highlight miR-4498 and miR-5689 as influencing factors in this process. Animal models of longevity have described the reduction in a ribonuclease involved in miRNA biogenesis, DICER, in aged animals compared to young controls. miRNA levels are significantly altered in aged animals [39]. Similarly, DICER has been shown to be decreased in animals with diet-induced obesity compared to lean controls with corresponding alterations in miRNA expression [40]. Previous studies have found alterations in expression levels of many miRNAs when fed a high-fat diet and during inflammatory states such as obesity [41]. Mice having a knockout (KO) of DICER in AT develop inflammation and IR, indicating miRNAs may play an active role in modulating inflammation [23]. If it is found that this translates to humans, this could potentially explain in part the increase in inflammatory-related conditions that are seen with obesity and aging. Recently, miR-4498 was linked to the expression of genes which are related to pro-inflammatory cytokines in patients with coronary artery disease [42]. The dysregulation of miR-4498 has also been observed in conditions associated with inflammation and immune dysfunction, such as major depressive disorder (MDD) and the development of preeclampsia [43]. miR-5689 has not been previously implicated in related conditions and has been the subject of very little research. However, while there are no known targets of miR-5680 included in the manually curated mirtarbase database (https://mirtarbase.cuhk.edu.cn/) of experimentally validated microRNA–target interactions, there are multiple predicted targets of miR-5689 in the miRDB database (https://mirdb.org/). These targets include inflammation-related genes, such as IL1RAP, an essential gene involved in IL-1-mediated inflammatory responses such as IL-1-dependent NF-κB activation, TAB3, involved in NF-κB activation and inflammatory cytokine production, and SOCS5, which regulates JAK/STAT inflammatory cytokine singality (gsea-MSigDBMSigDB.org). Additional predicted targets include insulin sensitivity-related genes, such as FOXO3, a transcription factor that regulates glucose metabolism, part of the FOXO family of genes that insulate insulin signaling, and HK1, a key enzyme involved in the first step of glycolysis, part of glucose metabolism (gsea-msigdb.org). Our study further illuminates the role of FO in modulating inflammation and IR, expanding on prior research linking miR-4498 as a possible biomarker for these conditions, or potentially as an active therapeutic target.

As result of dysregulated miRNA expression, we find that genes associated with acetyl-CoA hydrolase activity have been enriched. Elevated levels of acetyl-CoA, the substrate for this enzyme, are linked to IR and AT inflammation [31]. Acetyl-CoA disrupts normal metabolic pathways and interferes with insulin’s ability to effectively regulate glucose uptake. Reduction in acetyl-CoA through enhanced acetyl-CoA hydrolase activity may be a factor in the decreased inflammatory status and improved insulin sensitivity seen in our cohort after **ω**-3PUFA supplementation. Of further interest is that several gene sets involved in amino acid transport are shown to be enriched after ω-3PUFA supplementation and enhanced miRNA expression. It is well documented that increased circulating levels of branched-chained amino acids (BCAAs) are linked with people with obesity and IR [32]. In addition, sulfur-containing amino acids, methionine and cysteine have also been shown to be associated with obesity and IR where increased cysteine plasma level correlates with increased BMI [33] and methionine correlates with increased BCAA and the risk for the development of T2DM [34].

While these results are promising and shed light on an interesting relationship, we recognize the limitations of this study. We acknowledge that a larger cohort would provide greater power calculations to support our conclusions, and that the absence of a placebo-controlled comparator limits causal inference. However, this study was designed as a pilot, mechanistic, pre–post investigation aimed at identifying molecular changes associated with ω-3PUFA supplementation within individuals, rather than establishing definitive clinical efficacy. We further recognize that, by analyzing whole AT, there is the possibility that cell populations other than adipocytes, i.e., the stroma vascular fraction, may contribute to miRNA expression. It is well established, however, that AT adipocytes, which comprise the vast majority of cell types in AT, are a major source of circulating miRNA [23,44]. Additionally, due to the size of our cohort, we did not measure amino acid metabolites in our study participants, as the sample size would not provide adequate power to achieve significance; however, the enrichment of these pathways is notable and may be worthy of further research in larger cohorts. In order to thoroughly validate our conclusions as well as investigate their limits, it is necessary to conduct further studies in which cohort size is increased, and duration and dosage of treatment are varied. Future studies are also needed to explore the potential mechanisms of action of FO on the expression of miRNA and the improvement of insulin sensitivity.

Despite the limitations of this study, we are the first to describe miR-4498 and miR-5689 and their possible connection to the modulation of inflammation in humans. These miRNAs appear to play a role in the regulation of multiple pathways involved in modulating inflammation and ultimately insulin sensitivity. Our results suggest that the dysregulation of these miRNAs during obesity results in increased inflammation, which can be mediated through FO supplementation.

## Figures and Tables

**Table 1:** Primer sequences for AT qRT-PCR.

Gene	Primer Direction	Primer Sequences (5′ → 3′)
ACTB	Forward: Reverse:	AAACTGGAACGGTGAAGGTG AGAGAAGTGGGGTGGCTTTT
MIR-5689	Forward: Reverse:	AGCATACACCTGTAGTCCT GAACATGTCTGCGTATCTC
MIR-4498	Forward: Reverse:	TGGGCTGGCAGGGCAAGTG GAACATGTCTGCGTATCTC

**Table 2:** Clinical and anthropometric characteristics before and after 12 weeks ω-3 PUFA supplementation.

	Before ω-3 PUFA Supplementation	After ω-3 PUFA Supplementation	*p*-Value vs. Before ω-3 PUFA Supplementation
Sex (Female/Male)	9/3		
Age (years)	40.3 ± 2.6		
BMI (kg/m^2^)	40 ± 1.6	40 ± 1.6	n.s.
Body fat percentage (%)	46 ± 4.0	47 ± 4.0	n.s.
SBP (mmHg)	126 ± 2.6	114 ± 2.0	0.003
DBP (mmHg)	76 ± 2.0	73 ±1.8	n.s.
HR (BPM)	72.3 ± 4.5	70.5 ± 2.2	n.s.
A1c (%)	5.3 ± 0.1	5.4 ±0.1	n.s.

## Data Availability

Bioinformatic data supporting the conclusions of this article will be made available by the authors on request. Please email: defilippis.elena@mayo.edu for additional information.
